# Supplementary material for: Mapping the evolution of fertility support policies in China: A content and instrumental analysis
Source: PLoS One. 2025 Oct 9;20(10):e0332137. doi: 10.1371/journal.pone.0332137 (PMC12510515; doi:10.1371/journal.pone.0332137)
Supplement: S1 Appendix — (ZIP) [file pone.0332137.s001.zip › S1 Appendix. 226 original policy documents/93-劳动部关于公布中华人民共和国劳动保险条例实施细则修正草案的决定(FBM-CLI-4-55).doc]

劳动部关于公布中华人民共和国劳动保险条例实施细则修正草案的决定

**发布部门：** [劳动和社会保障部(含劳动部)(已撤销)](https://www.pkulaw.com/law/chl?Aggs.IssueDepartment=60212) [机构沿革](javascript:void(0))

**发布日期：**1953.01.26

**实施日期：**1953.01.01

**时效性：** [部分失效](https://www.pkulaw.com/law/chl?Aggs.TimelinessDic=05)

**效力级别：** [部门规范性文件](https://www.pkulaw.com/law/chl?Aggs.EffectivenessDic=XE0302)

**法规类别：** [工资福利与劳动保险](https://www.pkulaw.com/law/chl?Aggs.Category=08707)

**部分失效依据：**本篇法规中“有关女工人、女职员生育待遇的规定”已被[女职工劳动保护规定[失效]](javascript:TMP(3935,0,'chl'))废止

劳动部关于公布中华人民共和国劳动保险条例实施细则修正草案的决定
（一九五三年一月二十六日）

　　[中华人民共和国劳动保险条例](https://www.pkulaw.com/chl/96be87573ffd9605bdfb.html)实施细则草案已根据中央人民政府政务院公布的《关于[中华人民共和国劳动保险条例若干修正的决定](https://www.pkulaw.com/chl/c91c829fa1c5504ebdfb.html)》及修正的[劳动保险条例](https://www.pkulaw.com/chl/96be87573ffd9605bdfb.html)加以修改。兹决定仍作为草案公布试行并自一九五三年一月一日起有效。

部长　李立三

中华人民共和国劳动保险条例实施细则修正草案
（一九五三年一月二十六日劳动部公布试行）

　第一章　关于实施范围的规定

　　**第一条**　[中华人民共和国劳动保险条例](https://www.pkulaw.com/chl/96be87573ffd9605bdfb.html)(以下简称[劳动保险条例](https://www.pkulaw.com/chl/96be87573ffd9605bdfb.html))第[二条](https://www.pkulaw.com/chl/96be87573ffd9605bdfb.html#tiao_2)甲款关于“有工人职员100人以上”的规定，系指工厂、矿场本身的工人职员人数而言，其业务管理机关及附属单位人数不包括在内。在计算人数时，应包括工资制、供给制人员及学徒、临时工(临时性的建筑工人及搬运工人除外)、试用人员在内。

　　**第二条**　凡实行劳动保险的企业，其业务管理机关与附属单位，应与企业同时实行[劳动保险条例](https://www.pkulaw.com/chl/96be87573ffd9605bdfb.html)。“业务管理机关”系指其全部经费由业务收入或基本建设费或事业费内开支的机构而言。“附属单位”系指企业中所附设的有关业务及职工文化教育、福利等机构而言。

　　**第三条**　凡在实行劳动保险的企业附属单位内工作的工人职员及工会的工作人员，不由企业行政方面或资方直接支付工资者，其生育假期工资、病伤假期工资及因工死亡丧葬费由支付工资的单位付给，支付工资的单位应按各该单位工人职员的工资总额3％缴纳劳动保险金（原在企业工作的工会基层委员会脱离生产的委员，其劳动保险金可以免缴），交由企业行政方面或资方一并缴纳。工人职员及工会的工作人员医疗期间的医药费用，按[劳动保险条例](https://www.pkulaw.com/chl/96be87573ffd9605bdfb.html)的规定，由企业行政方面或资方负担。

　　**第四条**　在实行劳动保险的企业中的供给制人员，仍应按供给制的规定办理，不适用[劳动保险条例](https://www.pkulaw.com/chl/96be87573ffd9605bdfb.html)。厂矿企业的武装警卫人员，如系属于人民解放军建制的现役军人，仍应享受人民解放军的各种待遇，不适用[劳动保险条例](https://www.pkulaw.com/chl/96be87573ffd9605bdfb.html)。

　　**第五条**　不实行[劳动保险条例](https://www.pkulaw.com/chl/96be87573ffd9605bdfb.html)的企业订立劳动保险集体合同时，应根据[劳动保险条例](https://www.pkulaw.com/chl/96be87573ffd9605bdfb.html)的精神，及本企业、本产业或本行业的实际情况协商签订，并报请所在地劳动行政机关批准实行。集体合同中所规定的各项劳动保险费用，均由企业行政方面或资方负担。

　第二章　关于工资总额的规定

　　**第六条**　实行劳动保险的企业，按照[劳动保险条例](https://www.pkulaw.com/chl/96be87573ffd9605bdfb.html)第[八条](https://www.pkulaw.com/chl/96be87573ffd9605bdfb.html#tiao_8)的规定，缴纳劳动保险金时，工资总额的计算，应依照中央人民政府政务院财政经济委员会“关于[工资总额组成的规定](https://www.pkulaw.com/chl/759fc1f7e1f8a4d6bdfb.html)”办理。

　第三章　关于劳动保险待遇计算标准的规定

　　**第七条**　工人职员的工资按日或按月计算者，享受劳动保险待遇时，如按本人工资计算，均以该工人职员每日或每月所得工资为计算标准。

　　**第八条**　工人职员的工资按件计算者，享受劳动保险待遇时，如按本人工资计算，均以该工人职员最近三个月的每日平均工资为计算标准。如全部工作时间不满三个月者，应以该工人职员实际工作日的每日平均工资为计算标准。但病伤假期在七日以内者，得以上月份每日平均工资为计算标准。

　　**第九条**　领取因工残废补助费的工人职员退职或死亡时，在劳动保险基金项下领取以本人工资为计算标准的抚恤费、救济费或补助费时，其本人工资，应将所得工资与因工残废补助费合并计算。

　　**第十条**　学徒的本人工资低于该企业普通工人的最低工资者，以本人工资计算劳动保险费时，应改按该企业普通工人的最低工资计算，但其所得的劳动保险费，不得超过本人工资。

　第四章　关于因工负伤、残废、死亡待遇的规定

　　**第十一条**　工人职员在下列情况下负伤、残废或死亡时，应享受因工负伤、残废或死亡的待遇：
　　一、由于执行日常工作以及执行企业行政方面或资方临时指定或同意的工作；
　　二、在紧急情况下未经企业行政方面或资方指定而从事与企业有利的工作；
　　三、由于从事发明或技术改进的工作。
　　关于因工或非因工的确定，由工会小组据实报告工会基层委员会劳动保险委员会（以下简称劳动保险委员会）审查确定后，报请工会基层委员会通知企业行政方面或资方及工人职员本人或其供养直系亲属。如有不同意见时，应报请当地人民政府劳动行政机关迅速处理，但在未处理以前，应按工会基层委员会的通知办理。

　　**第十二条**　[劳动保险条例](https://www.pkulaw.com/chl/96be87573ffd9605bdfb.html)第[十二条](https://www.pkulaw.com/chl/96be87573ffd9605bdfb.html#tiao_12)丙款规定的残废审查委员会，应在市工会组织或产业工会地方组织领导下建立之，其人选由上述工会组织，劳动行政机关及卫生行政机关的代表三人至七人组成，以市工会组织或产业工会地方组织的代表为召集人，残废的工人职员所属企业劳动保险委员会的代表及主治医师得列席会议。只有个别企业实行[劳动保险条例](https://www.pkulaw.com/chl/96be87573ffd9605bdfb.html)的地方，不组织残废审查委员会，由该企业劳动保险委员会代行其职权。残废审查委员会的职权如下：
　　一、确定残废工人职员的残废状况；
　　二、每六个月检查残废者的残废状况一次，以确定其残废状况有无变更，但残废者请求审查残废状况时，得随时审查之。

　　**第十三条**　因工残废部分丧失劳动力尚能工作的工人职员，根据[劳动保险条例](https://www.pkulaw.com/chl/96be87573ffd9605bdfb.html)第[十二条](https://www.pkulaw.com/chl/96be87573ffd9605bdfb.html#tiao_12)乙款三项规定应领的因工残废补助费，按其残废后工资减少的数额付给：工资减少百分之十一至二十者，其因工残废补助费为残废前本人工资百分之十；工资减少百分之二十一至三十者，为残废前本人工资百分之二十；工资减少百分之三十以上者，一律补助残废前本人工资百分之三十。

　　**第十四条**　工人职员因工负伤在该企业医疗所、医院、特约医院、特约中西医师或转送之医院医疗终结后，必需安装假腿、假手、镶牙、补眼者，其所需费用，完全由企业行政方面或资方负担。

　　**第十五条**　工人职员因工残废部分丧失劳动力仍继续工作者，再次非因工残废仍能工作时，根据[劳动保险条例](https://www.pkulaw.com/chl/96be87573ffd9605bdfb.html)第[十二条](https://www.pkulaw.com/chl/96be87573ffd9605bdfb.html#tiao_12)乙款三项规定应领的因工残废补助费，应继续付给。再次非因工残废完全丧失劳动力不能工作而退职时，应按[劳动保险条例](https://www.pkulaw.com/chl/96be87573ffd9605bdfb.html)第[十三条](https://www.pkulaw.com/chl/96be87573ffd9605bdfb.html#tiao_13)丙款规定的待遇办理。

　第五章　关于疾病、非因工负伤、非因工残废待遇的规定

　　**第十六条**　工人职员疾病或非因工负伤停止工作连续医疗期间在六个月以内者，根据[劳动保险条例](https://www.pkulaw.com/chl/96be87573ffd9605bdfb.html)第[十三条](https://www.pkulaw.com/chl/96be87573ffd9605bdfb.html#tiao_13)乙款的规定，应由该企业行政方面或资方按下列标准支付病伤假期工资：本企业工龄不满二年者，为本人工资百分之六十；已满二年不满四年者，为本人工资百分之七十；已满四年不满六年者，为本人工资百分之八十；已满六年不满八年者，为本人工资百分之九十；已满八年及八年以上者，为本人工资百分之一百。

　　**第十七条**　工人职员疾病或非因工负伤停止工作连续医疗期间超过六个月时，根据[劳动保险条例](https://www.pkulaw.com/chl/96be87573ffd9605bdfb.html)第[十三条](https://www.pkulaw.com/chl/96be87573ffd9605bdfb.html#tiao_13)乙款的规定，病伤假期工资停发，改由劳动保险基金项下，按月付给疾病或非因工负伤救济费，其标准如下：本企业工龄不满一年者，为本人工资百分之四十；已满一年未满三年者，为本人工资百分之五十；三年及三年以上者，为本人工资百分之六十。此项救济费付至能工作或确定为残废或死亡时止。

　　**第十八条**　工人职员的本人工资低于该企业的平均工资者，领取疾病或非因 工负伤救济费时，如其所得救济费数额低于该企业的平均工资百分之四十，应按平均工资百分之四十发给，但不得高于本人工资。

　　**第十九条**　工人职员因工残废完全丧失劳动力而退职者及退职养老者，本人仍得按[劳动保险条例](https://www.pkulaw.com/chl/96be87573ffd9605bdfb.html)第[十三条](https://www.pkulaw.com/chl/96be87573ffd9605bdfb.html#tiao_13)甲款的规定，继续享受疾病医疗待遇至死亡时止。

　　**第二十条**　工人职员疾病或非因工负伤医疗终结，确定为残废完全丧失劳动力退职后，除领取非因工残废救济费、本人死亡时的丧葬补助费及供养直系亲属救济费外，其他劳动保险待遇应停止享受。

　　**第二十一条**　工人职员疾病或非因工负伤需要停止工作医疗者，须经负责医疗机关提出证明，如当时无法取得上项证明，须由工会小组长或小组劳动保险干事证明，始得享受劳动保险待遇。病愈复工时，应取得负责医疗机关提出能工作的证明。

　第六章　关于死亡待遇的规定

　　**第二十二条**　工人职员因工死亡时或因工残废退职后死亡时，根据[劳动保险条例](https://www.pkulaw.com/chl/96be87573ffd9605bdfb.html)第[十四条](https://www.pkulaw.com/chl/96be87573ffd9605bdfb.html#tiao_14)甲款的规定，由企业行政方面或资方发给本企业的平均工资三个月作为丧葬费，并按下列规定，由劳动保险基金项下每月付给供养直系亲属抚恤费：其供养直系亲属一人者，为死者本人工资百分之二十五；二人者，为死者本人工资百分之四十；三人或三人以上者，为死者本人工资百分之五十。此项抚恤费付至受供养者失去受供养的条件时止（供养条件见第十一章的规定）。

　　**第二十三条**　工人职员因病或非因工负伤死亡时、退职养老后死亡时或非因工残废完全丧失劳动力退职后死亡时，根据[劳动保险条例](https://www.pkulaw.com/chl/96be87573ffd9605bdfb.html)第[十四条](https://www.pkulaw.com/chl/96be87573ffd9605bdfb.html#tiao_14)乙款的规定，除由劳动保险基金项下付给本企业的平均工资二个月作为丧葬补助费外，并按下列规定由劳动保险基金项下一次付给供养直系亲属救济费：其直系亲属一人者，为死者本人工资六个月；二人者。为死者本人工资九个月；三人或三人以上者，为死者本人工资十二个月。

　　**第二十四条**　工人职员因工死亡或因工残废完全丧失劳动力，其供养直系亲属具有工作能力而该企业需人工作时，行政方面或资方应尽先录用；受其供养的子、女、弟、妹有入该企业所办学校就读的权利。

　　**第二十五条**　全家有两人或两人以上在实行劳动保险的企业内工作，其共同供养的直系亲属死亡时，丧葬补助费应由其中一人领取，不得重领。

　第七章　关于养老待遇的规定

　　**第二十六条**　工人职员退职养老时，应由劳动保险基金项下按月付给退职养老补助费：本企业工龄已满五年未满十年者，付给本人工资百分之五十；已满十年未满十五年者，付给本人工资百分之六十；已满十五年及十五年以上者，付给本人工资百分之七十。付至死亡时止。

　　**第二十七条**　合于[劳动保险条例](https://www.pkulaw.com/chl/96be87573ffd9605bdfb.html)第[十五条](https://www.pkulaw.com/chl/96be87573ffd9605bdfb.html#tiao_15)养老规定的工人职员，因企业需要留其继续工作者，除工资照发外，另由劳动保险基金项下按月付给在职养老补助费：本企业工龄已满五年不满十年者，付给本人工资百分之十；已满十年不满十五年者，付给本人工资百分之十五；已满十五年及十五年以上者，付给本人工资百分之二十。

　　**第二十八条**　领取在职养老补助费的工人职员，退职养老时，其在职养老补助费，不应计算在本人工资之内。

　　**第二十九条**　工人职员如曾从事井下矿工或固定在华氏三十二度以下低温工作场所或华氏一百度以上高温工作场所的工作时间，前后合计达十年，或直接从事铅、汞、砒、磷、酸及其他化学、兵工工业的有害身体健康工作时间，前后合计达八年，现虽未从事上述各项工作，仍得按[劳动保险条例](https://www.pkulaw.com/chl/96be87573ffd9605bdfb.html)第[十五条](https://www.pkulaw.com/chl/96be87573ffd9605bdfb.html#tiao_15)丙、丁两款的规定，享受退职养老待遇。

　　**第三十条**　工人职员在该企业实行劳动保险后，由于年老力衰调动工作，其工资降低至该企业的平均工资以下者，在退职养老时，其退职养老补助费，应按该企业的平均工资计算。

　第八章　关于生育待遇的规定

　　**第三十一条**　[劳动保险条例](https://www.pkulaw.com/chl/96be87573ffd9605bdfb.html)第[十六条](https://www.pkulaw.com/chl/96be87573ffd9605bdfb.html#tiao_16)规定的产假(不论正产或小产)，应包括星期日及法定假日在内，不再补假。

　　**第三十二条**　女工人女职员或男工人男职员之妻生育，如系双生或多生时，其生育补助费应按其所生子女人数，每人发给八万元。

　　**第三十三条**　夫妻同在一个或分别在两个实行劳动保险的企业内工作者，生育补助费应由妻领取，其夫不得重领。

　　**第三十四条**　女工人女职员怀孕不满七个月小产的产假，为三十日以内，但最少应为二十日。

　　**第三十五条**　女工人女职员生育，如该企业医疗所、医院、特约医院、特约医师无法接生时，其接生费用，亦由企业行政方面或资方负担。

　第九章　关于临时工、季节工及试用人员劳动保险待遇的规定

　　**第三十六条**　实行劳动保险的企业的临时工、季节工及试用人员，其劳动保险待遇暂定为下列各项：
　　一、因工负伤医疗期间待遇与一般工人职员同。因工负伤医疗终结，确定为残废后完全丧失劳动力而退职者，由劳动保险基金项下，一次付给因工残废抚恤费，其数额为本人工资十二个月；部分丧失劳动力尚能工作者，由企业行政方面或资方分配适当工作。
　　二、患病或非因工负伤的医疗期间以三个月为限，其医疗待遇与一般工人职员同。停工医疗期间，在三个月以内者，由企业行政方面或资方按月发给病伤假期工资，其数额为本人工资百分之五十；满三个月尚未痊愈者，由劳动保险基金项下一次付给本人工资三个月的疾病或非因工负伤救济费。
　　三、因工死亡时，由企业行政方面或资方发给丧葬费，其数额为本企业的平均工资三个月，另由劳动保险基金项下一次付给供养直系亲属抚恤费：其供养直系亲属一人者，发给本人工资六个月；二人者，发给本人工资九个月；三人或三人以上者，发给本人工资十二个月。
　　四、疾病或非因工负伤死亡时，由劳动保险基金项下付给丧葬补助费，其数额为本企业的平均工资两个月；另由劳动保险基金项下一次付给供养直系亲属救济费，其数额为本人工资三个月。
　　五、怀孕及生育的女工人女职员，其怀孕检查费、接生费、生育补助费及生育假期与一般女工人女职员同；产假期间由企业行政方面或资方发给产假工资，其数额为本人工资百分之六十。

　　**第三十七条**　在本细则公布前，各企业或所属产业或行业原定有关临时工、季节工及试用人员劳动保险待遇的标准高于本细则第三十六条的规定者，可仍按原规定支付。

　第十章　关于工龄的规定

　　**第三十八条**　一般工龄系指工人职员以工资收入为生活资料之全部或主要来源的工作时间而言。在计算一般工龄时，应包括本企业工龄在内。

　　**第三十九条**　本企业工龄应以工人职员在本企业连续工作的时间计算之，如曾离职，应自最后一次回本企业工作之日算起。但有下列情况之一者，不在此限：
　　一、凡经企业管理机关、企业行政方面或资方调动工作者，其调动前后的本企业工龄，均应连续计算。但解放前确因业务需要调动工作具有确实证明者，其本企业工龄，始得连续计算。
　　二、解放前在本企业工作，曾经被迫离职又回本企业工作者，如有确实证明，经工会小组讨论通过后，并经劳动保险委员会批准，其离职前与回本企业后的工作时间，可合并作本企业工龄计算。
　　三、解放后经企业管理机关、企业行政方面或资方调派国内外学习者，其学习期间及调派前后的本企业工龄，应连续计算。解放前经企业管理机关、企业行政方面或资方调派国内外学习业务者，如有确实证明，除学习期间不计工龄外，其调派前与回本企业后的本企业工龄，得合并计算。
　　四、解放后因企业停工歇业或缩减生产，其工人职员经企业管理机关调派至其他企业工作者，其调派前后的本企业工龄，应连续计算。被遣散的工人职员在该企业复工复业或扩大生产时，仍回本企业工作者，其遣散前与复工后的本企业工龄，应合并计算。
　　五、企业经转让、改组或合并，原有工人职员仍留企业工作者，其转让、改组或合并前后的本企业工龄，应连续计算。
　　六、因工负伤停止工作医疗期间，应全部作为本企业工龄计算。
　　七、疾病或非因工负伤停止工作医疗期间，在六个月以内者，得连续作本企业工龄计算；超过六个月病愈后，仍回原企业工作者，除超过六个月的期间不算工龄外，其前后本企业工龄，应合并计算。
　　八、在敌伪及国民党反动统治下为反对其统治压迫而被迫离职，在离职期间被敌伪及国民党政府监禁仍继续斗争者，其在监禁期间及离职前与复职后或转入其他企业工作的本企业工龄，均应连续计算。

　　**第四十条**　转入企业工作的专门从事革命工作者及革命军人，其从事革命工作的年限及军龄，均应作本企业工龄计算。

　　**第四十一条**　凡工人职员在敌伪及国民党反动统治时期，充任下列职务之一者，其从事该项职务的时间，一律不作工龄计算：
　　一、把头、监工、厂警、矿警等有压迫剥削行为者。
　　二、敌伪及国民党军队宪兵中的官兵，警察中的警官警察，敌伪及国民党政府机关中的官吏，但不包括企业机关中的人员。
　　三、国民党区分部委员以上，三民主义青年团分队长以上，青年党区党部委员以上及民主社会党区支部委员以上的人员，反动会道门主要负责人。

　　**第四十二条**　凡被剥夺政治权利者，其被剥夺政治权利期间，不作工龄计算。因反革命罪行而被剥夺政治权利者，其本企业工龄，应自恢复政治权利之日算起；因其他犯罪行为而被剥夺政治权利者，其被剥夺政治权利前与恢复政治权利后的本企业工龄，应合并计算。

　　**第四十三条**　[劳动保险条例](https://www.pkulaw.com/chl/96be87573ffd9605bdfb.html)第[十五条](https://www.pkulaw.com/chl/96be87573ffd9605bdfb.html#tiao_15)丙、丁两款关于折算一般工龄及本企业工龄的规定，在计算各项劳动保险待遇时，均同样适用。

　　**第四十四条**　学徒在本企业学习期间，应作本企业工龄计算，临时工、试用人员转为正式工人职员时，其本企业工龄，应自入该企业工作之日算起。

　第十一章　关于供养直系亲属的规定

　　**第四十五条**　工人职员的直系亲属。其主要生活来源，系依靠工人职员供给，并合于下列各款规定之一者，均得列为该工人职员的供养直系亲属，享受劳动保险待遇：
　　一、祖父、父、夫年满六十岁或完全丧失劳动力者；
　　二、祖母、母、妻未从事有报酬的工作者；
　　三、子女（包括养子女，前妻或前夫所生子女，非婚生子女）、弟妹（包括同父异母或同母异父的弟妹）年未满十六岁；
　　四、孙子女年未满十六岁，其父死亡或完全丧失劳动力，母未从事有报酬的工作者。

　　**第四十六条**　工人职员自幼依靠他人抚养长大，现抚养人男年满六十岁或完全丧失劳动力，女未从事有报酬的工作，须依靠工人职员本人供养且共同居住者，得以供养直系亲属论。

　　**第四十七条**　工人职员因工死亡后，其遗腹子得列为供养直系亲属。

　　**第四十八条**　工人职员的供养直系亲属，如不与工人职员同在一处居住，须取得其所在地政府机关证明，确系依靠工人职员供养，方得列为供养直系亲属。

　第十二章　关于医疗机构的规定

　　**第四十九条**　实行劳动保险的企业，已设立医疗机构者，应根据必要与可能的情况，充实设备，并应建立健全制度；未设立医疗机构者，应单独或联合设立医疗所或医院；如因条件限制不能设立，应有特约医院或特约中西医师，为病伤工人职员负责医治。所有有关医疗所、医院、特约医院、特约中西医师的一切费用，均由企业行政方面或资方负担。

　第十三章　关于疗养所、业余疗养所、托儿所的规定

　　**第五十条**　实行劳动保险的企业，应根据工人职员的需要及企业经济情况，单独或联合其他企业设立疗养所、业余疗养所、营养食堂。疗养所、业余疗养所、营养食堂的房屋设备、工作人员的工资及一切经常费用，完全由企业行政方面或资方负担。休养员及营养员的伙食费，在劳动保险基金支付各项补助费、救济费、抚恤费后有剩余时得给予补助，最多不得超过伙食费的二分之一，如因身体衰弱或经济确实困难负担不起伙食费者得酌量提高其补助费，但不得超过伙食费的三分之二。各企业工会基层委员会，应按期编制伙食补助费的预算，送掌管调剂金的上级工会组织批准后执行。

　　**第五十一条**　实行劳动保险的企业的女工人女职员，有四周岁以内的子女二十人以上，工会基层委员会与企业行政方面或资方协商单独或联合其他企业设立托儿所(如尚未具备设立托儿所条件，而有哺乳婴儿五个以上须设立哺乳室)。其房屋设备、工作人员的工资及一切经常费用，完全由企业行政方面或资方负担，托儿饮食费由托儿父母负担，如托儿父母经济确有困难者，得由劳动保险基金项下予以补助，但对每个儿童的补助不得超过托儿饮食费的三分之一。

　第十四章　关于优异劳动保险待遇的规定

　　**第五十二条**　合于[劳动保险条例](https://www.pkulaw.com/chl/96be87573ffd9605bdfb.html)第[十九条](https://www.pkulaw.com/chl/96be87573ffd9605bdfb.html#tiao_19)的规定。享受优异劳动保险待遇的工人职员因工死亡时，其供养直系亲属抚恤费，依下列规定由劳动保险基金项下按月付给：其供养直系亲属一人者，为死者本人工资百分之三十；二人者，为死者本人工资百分之四十五；三人及三人以上者，为死者本人工资百分之六十。此项抚恤费付至受供养者失去受供养的条件时止。

　　**第五十三条**　合于[劳动保险条例](https://www.pkulaw.com/chl/96be87573ffd9605bdfb.html)第[十九条](https://www.pkulaw.com/chl/96be87573ffd9605bdfb.html#tiao_19)的规定，享受优异劳动保险待遇的工人职员退职养老时，按月付给退职养老补助费：本企业工龄已满五年未满十年者，为本人工资百分之六十；已满十年未满十五年者，为本人工资百分之七十；已满十五年及十五年以上者，为本人工资百分之八十。此项补助费付至死亡时止。合于养老规定的工人职员，因企业工作需要，留其继续工作时，除工资照发外，另由劳动保险基金项下，按月付给在职养老补助费：本企业工龄已满五年未满十年者，为本人工资百分之二十；已满十年未满十五年者，为本人工资百分之二十五；已满十五年及十五年以上者，为本人工资百分之三十。此项补助费付至退职养老或死亡时止。

　第十五章　关于劳动保险各项费用申请与支付手续的规定

　　**第五十四条**　凡在劳动保险基金项下支付的各项抚恤费、补助费与救济费，均由工人职员或供养直系亲属向劳动保险委员会申请领取，其程序规定如下：
　　一、开始申请时，应据实填写申请书，如需证明，应将证明文件一并提交工会小组劳动保险干事或工会小组长召开小组会议讨论，审查所填各项是否属实，并将审查意见填入申请书“工会小组意见”栏内，签名盖章后，送交劳动保险委员会。
　　二、劳动保险委员会接到申请书，查对劳动保险登记卡片后，由劳动保险委员会主任（必要时可召开劳动保险委员会讨论）在申请书上批准应享受的劳动保险待遇及其计算标准，并填具付款凭单，连同申请书一并交由申请人持往劳动保险会计处取款，如系按月领取者，应于批准申请书时发给领取证。事后须在劳动保险委员会定期会议上汇报。
　　三、劳动保险委员会，根据工作需要，可授权车间劳动保险委员会，依照[劳动保险条例](https://www.pkulaw.com/chl/96be87573ffd9605bdfb.html)及其实施细则的规定，审查批准工人职员的劳动保险待遇。
　　四、劳动保险会计付款后，应在申请书上注明支付金额及日期、签名盖章，并将申请书退还劳动保险委员会负责保管。
　　五、按月领取劳动保险待遇者，应在每月领款前，将领取证送经劳动保险委员会主任签名或盖章并填具付款单后，持往劳动保险会计处领款，如其本人或供养直系亲属不在当地，须附所在地政府机关开具的证明文件，连同领取证一并寄交劳动保险委员会申请汇付，其汇费及邮费，均由劳动保险基金项下支付。

　　**第五十五条**　劳动保险委员会付给的抚恤费、补助费、救济费，按月付给者，至迟应于每月月底付给，如领款人不在当地居住，劳动保险委员会可按季度付给（三个月付一次）。一次发给者，至迟应于批准后五日内发给。

　　**第五十六条**　由企业行政方面或资方直接支付的病伤、生育假期工资及因工死亡丧葬费，于每月发放后，应将领款人姓名、支付标准、支付金额按费别造表，送交劳动保险委员会备查。

　　**第五十七条**　因工死亡丧葬费、非因工死亡丧葬补助费，由死者供养直系亲属或直系亲属申请领取，如无上述亲属或亲属不在当地，得由工会基层委员会代为领取，负责办理丧葬事宜。

　　**第五十八条**　凡实行劳动保险的企业，如迁移至其他地区，其不能随企业迁移的工人职员按月领取的抚恤费、补助费与救济费，由省市工会或产业工会全国委员会在调剂金中继续支付；工人职员病伤尚未痊愈者，应继续予以治疗，由企业行政方面或资方按照[劳动保险条例](https://www.pkulaw.com/chl/96be87573ffd9605bdfb.html)第[十二条](https://www.pkulaw.com/chl/96be87573ffd9605bdfb.html#tiao_12)甲款或第[十三条](https://www.pkulaw.com/chl/96be87573ffd9605bdfb.html#tiao_13)甲、乙两款的规定，汇发医药费及医疗期间工资。

　　**第五十九条**　凡实行劳动保险的企业歇业时，其工人职员按月领取的抚恤费、补助费与救济费，由省市工会或产业工会全国委员会在调剂金中继续支付，工人职员病伤尚未痊愈者，应在歇业时，由企业行政方面或资方与工会基层委员会协商处理之。

　　**第六十条**　凡实行劳动保险的企业，如经转让、改组或合并，其全部劳动保险业务，应由接办者继续办理，所有对工人职员应继续支付的各项劳动保险费用，不得中止付给。

　　**第六十一条**　领取抚恤费、补助费与救济费的工人职员或供养直系亲属，如被剥夺政治权利，应即停止发给，政治权利恢复时，经其本人提出证明，送请劳动保险委员会批准，仍得继续领取，但停发期间的劳动保险待遇，不予补发。

　　**第六十二条**　在实行劳动保险的企业内工作的工人职员，经企业管理机关调至其他实行劳动保险的企业内工作时，由劳动保险委员会将劳动保险登记卡片及有关劳动保险文件寄交调用企业，劳动保险委员会于工人职员到达后办理登记，继续享受劳动保险待遇。如在调用途中发生病、伤、残废或死亡时，应由调用企业按照[劳动保险条例](https://www.pkulaw.com/chl/96be87573ffd9605bdfb.html)的规定办理。至于由已实行劳动保险的企业调到未实行劳动保险的企业内工作时，其有关劳动保险待遇事项，应按调用单位的现有规定办理。

　　**第六十三条**　工人职员或其供养直系亲属对劳动保险待遇的决定、核算及支付，与企业行政方面或资方及劳动保险委员会有不同意见时，可提请上级工会组织处理，或向当地人民政府劳动行政机关提出申诉。

　　**第六十四条**　领取各项抚恤费、补助费与救济费的工人职员或其供养直系亲属，如其领取条件有变更或失去领取条件时，应即报告劳动保险委员会，如有虚报匿报情形时，经查出后，劳动保险委员会得按情节轻重，给以公开批评或停止享受各项劳动保险待遇的权利一个月至三个月，并追还匿报虚报所领取的金额。

　　**第六十五条**　凡按月领取的抚恤费、补助费与救济费的计算标准有变动时，由劳动保险委员会在领取证上批注；其种类有变更时，将原领取证交回注销，并由申请人另填申请书申请之。

　第十六章　关于劳动保险金保管收支的规定

　　**第六十六条**　属于铁路、邮电、兵工及海员工会全国委员会的工会基层委员会，每月应将劳动保险基金的剩余部分，转入各该产业工会全国委员会户内，作为调剂金；不属于上述产业工会全国委员会的工会基层委员会，每月应将劳动保险基金的剩余部分，转入直属上级的省、市工会组织户内，作为调剂金。纺织、煤矿及其他产业工会，如具备自行掌管调剂金的条件时，经中华全国总工会批准，即可自行掌管调剂金。

　　**第六十七条**　凡掌管调剂金的产业工会全国委员会，如授权其地方组织掌管调剂金调用时，应根据各该产业的具体情况，制定详细办法，报告中华全国总工会及中央人民政府劳动部备案。

　　**第六十八条**　滞纳金由工会基层委员会转入劳动保险基金项下，其用途与劳动保险基金同。

　　**第六十九条**　各企业会计部门，应依照劳动保险基金会计制度的规定，单独设立账簿，记载劳动保险基金的收支事项。此项工作，由企业会计部门办理，并对劳动保险委员会负责。

　　**第七十条**　各企业在开始实行劳动保险时，工会基层委员会，应按该企业实有人数，报告产业工会全国委员会或省、市工会组织，并编制劳动保险登记卡片印制费预算，报请中华全国总工会核发，由劳动保险总基金内支付。工会基层组织办理劳动保险业务所需的申请书、领取证的纸张印刷费，应报请掌管调剂金的省、市工会组织及产业工会全国委员会核发，由调剂金内支付。各级工会组织所需的统计表的纸张印刷费、宣传费、办公费及其他开支，由工会工作费内支付。企业会计部门办理劳动保险基金会计人员的工资、办公费用、传票、账簿、报表及其他经常开支，均由企业行政方面或资方负担。

　第十七章　关于劳动保险业务的监督与检查的规定

　　**第七十一条**　各大行政区工会组织，每三个月向所在地大行政区劳动行政机关、中央人民政府劳动部及中华全国总工会报送的劳动保险基金收支报告，应按季度编造，即以每年一、二、三月为第一季度，四、五、六月为第二季度，七、八、九月为第三季度，十、十一、十二月为第四季度。如开始报告的月份在季度开始之第二个月或第三个月时，亦应按照本条规定办理，不得跨季度或年度编造。

　　**第七十二条**　经费审查委员会对于劳动保险基金收支账目，有随时审查之权，发现错误，可立即提出意见，企业的会计部门或劳动保险委员会应迅速答复或改正，如逾十日仍未答复或处理不当，其错误系属于企业会计部门者，可提请企业行政方面或资方处理，必要时得提请当地人民政府劳动行政机关追究；如错误系属劳动保险委员会者，得提请工会基层委员会或上级工会组织处理之。

　　**第七十三条**　工人职员对劳动保险基金的收支及企业行政方面或资方直接支付的各项劳动保险费用，发现疑问或错误时，可报告工会基层委员会处理，如情节重大，得越级报告上级工会组织或当地人民政府劳动行政机关处理之。

　　**第七十四条**　各企业会计部门，应按月编造由企业行政方面或资方直接支付的劳动保险费用月报表，送经费审查委员会审查后，报请工会基层委员会核转省、市工会组织和产业工会全国委员会及当地人民政府劳动行政机关。直接支付劳动保险费用月报表应设下列各项目：
　　一、因工负伤医疗期间工资；
　　二、疾病及非因工负伤医疗期间工资；
　　三、产假期间工资；
　　四、因工死亡丧葬费；
　　五、医药费及医务经费；
　　六、疗养所经费；
　　七、业余疗养所经费；
　　八、营养食堂经费；
　　九、托儿所及哺乳室经费；
　　十、其他支出。

　　**第七十五条**　各级人民政府劳动行政机关，对所辖地区内各企业的行政方面或资方及各级工会组织的劳动保险业务，应根据[劳动保险条例](https://www.pkulaw.com/chl/96be87573ffd9605bdfb.html)第[三十条](https://www.pkulaw.com/chl/96be87573ffd9605bdfb.html#tiao_30)的规定，实施监督与检查，并得随时检查各企业实施劳动保险的状况，调阅有关劳动保险的各项账册、报表及其他有关文件，如发现有违反[劳动保险条例](https://www.pkulaw.com/chl/96be87573ffd9605bdfb.html)事情，应即予以纠正，或报请上级劳动行政机关处理，必要时，并得移送司法机关依法办理。

　第十八章　附　则

　　**第七十六条**　合于[劳动保险条例](https://www.pkulaw.com/chl/96be87573ffd9605bdfb.html)第[二条](https://www.pkulaw.com/chl/96be87573ffd9605bdfb.html#tiao_2)规定的企业，应由其工会基层委员会与行政方面或资方，联名向当地省、市人民政府劳动行政机关申请实行劳动保险经登记核准后，由工会基层委员会用书面报告上级工会组织逐级转报中华全国总工会备案。但铁路、邮电、兵工工会的全国委员会，得各自会同行政方面，联名向中央人民政府劳动部申请实行劳动保险，经登记核准后，立即用书面报告中华全国总工会备案，其所属的企业单位，不再向当地劳动行政机关申请登记。

　　**第七十七条**　各省、市人民政府劳动行政机关，应将各该地区业经批准实行劳动保险的企业，报告上级劳动行政机关逐级转报中央人民政府劳动部备案。

　　**第七十八条**　[劳动保险条例](https://www.pkulaw.com/chl/96be87573ffd9605bdfb.html)所称“省、市工会”的市，系指中央人民政府直辖市、大行政区行政委员会直辖市及省人民政府直辖市而言。

　　**第七十九条**　本细则由中央人民政府劳动部公布施行，如有未尽事宜，得由中央人民政府劳动部随时补充修改之。
　　备注：
　　1.有关劳动保险基金项下支付的各项费用，根据一九七三年五月十五日财政部财企第41号文的规定，改在营业外支付；
　　2.有关养老待遇和因工、非因工残废完全丧失劳动能力的待遇的规定，与一九七八年六月二日国务院颁发的《国务院关于安置老弱病残干部的暂行办法》和《国务院关于工人退休、退职的暂行办法》中的规定有抵触时，应按一九七八年两个暂行办法的规定执行；
　　3.根据国务院一九七一年十一月三十日（71）国发文91号文和国家计委劳动局一九七三年六月十一日（73）计劳业字57号文的规定，临时工因工死亡和因工致残完全丧失劳动能力的待遇均按固定工的待遇执行；
　　4.第六条“关于[工资总额组成的规定](https://www.pkulaw.com/chl/759fc1f7e1f8a4d6bdfb.html)”，应改按一九五五年五月二十一日国务院（55）国秘云字第103号文批准的“中华人民共和国国家统计局关于工资总额组成的暂行规定”和（78）财企字第641号、（79）财企字第657号文的规定办理；
　　5.第三十二条“万元”为旧币。

© 北大法宝：（[www.pkulaw.com](https://www.pkulaw.com/)) 专业提供法律信息、法学知识和法律软件领域各类解决方案。北大法宝为您提供丰富的参考资料，正式引用法规条文时请与标准文本核对。
欢迎查看所有[产品和服务](https://www.pkulaw.com/helps/2.html)。法宝快讯：[如何快速找到您需要的检索结果？](https://www.pkulaw.com/helps/70.html)[法宝](https://www.pkulaw.com/helps/69.html)V6有何新特色？

扫描二维码阅读原文

**原文链接：**<https://www.pkulaw.com/chl/facadd9fc5e060bcbdfb.html>
